# Anxiety, Depression, and General Psychological Distress in Patients with Coronary Slow Flow

**DOI:** 10.5935/abc.20150092

**Published:** 2015-10

**Authors:** Mehmet Baran Karataş, Ebru Şahan, Kazım Serhan Özcan, Yiğit Çanga, Barış Güngör, Tolga Onuk, Göktürk İpek, Yasin Çakıllı, Emre Arugaslan, Osman Bolca

**Affiliations:** 1 Siyami Ersek Thoracic and Cardiovascular Surgery Center, Training and Research Hospital Department of Cardiology; 2 Haydarpaşa Numune Training and Research Hospital Department of Psychiatry; 3 Derince Training and Research Hospital Department of Cardiology; 4 Kartal Yavuz Selim Hospital Department of Cardiology

**Keywords:** Coronary Circulation, Depression, Anxiety Disorders, Stress, Psychological, Coronary Artery Disease / psychology

## Abstract

**Background:**

The relationship between psychiatric illness and heart disease has been frequently discussed in the literature. The aim of the present study was to investigate the relationship between anxiety, depression and overall psychological distress, and coronary slow flow (CSF).

**Methods:**

In total, 44 patients with CSF and a control group of 50 patients with normal coronary arteries (NCA) were prospectively recruited. Clinical data, admission laboratory parameters, and echocardiographic and angiographic characteristics were recorded. Symptom Checklist 90 Revised (SCL-90-R), Beck Depression Inventory (BDI), and Beck Anxiety Inventory (BAI) scales were administered to each patient.

**Results:**

The groups were comparable with respect to age, sex, and atherosclerotic risk factors. In the CSF group, BAI score, BDI score, and general symptom index were significantly higher than controls (13 [18.7] vs. 7.5 [7], p = 0.01; 11 [14.7] vs. 6.5 [7], p = 0.01; 1.76 [0.81] vs. 1.1[0.24], p = 0.01; respectively). Patients with CSF in more than one vessel had the highest test scores. In univariate correlation analysis, mean thrombolysis in myocardial infarction (TIMI) frame counts were positively correlated with BAI (r = 0.56, p = 0.01), BDI (r = 0.47, p = 0.01), and general symptom index (r = 0.65, p = 0.01). The psychiatric tests were not correlated with risk factors for atherosclerosis.

**Conclusion:**

Our study revealed higher rates of depression, anxiety, and overall psychological distress in patients with CSF. This conclusion warrants further studies.

## Introduction

The effect of psychiatric disorders on the incidence and progression of cardiovascular diseases has been investigated in previous studies^1^. Concomitant depression is associated with an increased risk of cardiac morbidity and mortality after an acute myocardial infarction (MI) and coronary revascularization procedures^2,3^.

Coronary slow flow (CSF) is a relatively common angiographic phenomenon that is characterized by slow progression of a contrast agent through the coronary arteries in the absence of any stenosis^4^. Functional and morphological abnormalities in the microvasculature, endothelial dysfunction, raised inflammatory markers, occult atherosclerosis, and anatomical factors of epicardial arteries have all been implicated in the pathogenesis of CSF^5^. However, little is known about the relationship between CSF and psychiatric disorders. Thus, in this study, we aimed to investigate the correlation of CSF with anxiety, depression, and general psychiatric status.

## Methods

### Study Protocol

We prospectively enrolled 44 consecutive patients with CSF who had undergone diagnostic coronary angiography (CAG) between January 2014 and March 2014 in Siyami Ersek Training and Research Hospital. The control group consisted of 50 consecutive patients with normal coronary arteries who had undergone CAG between January 2014 and March 2014. Indications of coronary angiographies were determined with positive results of myocardial ischemia in noninvasive myocardial imaging and typical angina pectoris. All patients were assessed for demographic features, cardiovascular risk factors, laboratory parameters, and medications. The local ethics committee approved the study protocol and written informed consent was obtained from the study participants according to the Declaration of Helsinki.

Exclusion criteria included the following: refusal to participate in the study, known coronary artery disease (CAD), acute coronary syndrome, left ventricular (LV) dysfunction (defined as LV ejection fraction [LVEF] < 50%), severe valvular heart disease, rhythm other than sinus, end-stage renal or hepatic dysfunction, severe chronic obstructive pulmonary disease, systemic diseases, subjects receiving medical treatment for any type of psychiatric disorder, insufficient cooperation, presence of coronary artery stenosis > 20%, or any type of congenital coronary abnormality (such as myocardial bridging and coronary fistulas).

Hypertension (HT) was defined as the use of antihypertensive drugs or initial blood pressure over 140/90 mmHg. Diabetes mellitus (DM) was defined as the use of antidiabetic drugs or fasting plasma glucose levels of > 126 mg/dL. Hyperlipidemia was defined as total serum cholesterol levels > 240 mg/dL. Smoking status was defined as current tobacco use. Body mass index (BMI) was calculated by dividing weight into the square of height.

Venous blood samples were taken from the antecubital vein and collected in calcium EDTA tubes, and were then studied by an auto-analyser (Cell-dyn 3700 Abbott, USA) on the day of CAG. Transthoracic echocardiography (Vivid 3 system, General Electric, made in Norway) was performed prior to CAG to detect LV functions and valvular heart disease. LVEF was measured using the modified Simpson method.

### Coronary angiography and documentation of coronary slow flow

Selective CAG was performed by the femoral approach using the Judkins technique. Angiographic images were obtained by the Simens Axiom 792 Axa Angiographic System. Multiple views were obtained with visualization of the left anterior descending (LAD) and left circumflex coronary arteries in at least four projections, and the right coronary artery in at least two projections. CAGs were recorded at 15 frames per second and recorded on compact discs in DICOM format. Angiograms were interpreted by two cardiologists and thrombolysis in myocardial infarction (TIMI) frame counts (TFC) were calculated for each coronary artery^6^. TFC were defined as the number of cine frames required for the contrast agent to reach standardized distal coronary landmarks as described by Gibson et al^6^. TFC for the left anterior descending artery were divided by 1.7 to obtain the corrected TFC (cTFC). Since the most frequently standardized filming rate is 30 frames per second, the TFCs were multiplied by 2. The subjects with a TFC greater than two standard deviations (SD) above the normal range were considered to have CSF^6^. Total TFC was defined as the sum of TFC in three major epicardial vessels. Mean TFC was calculated by dividing the total TFC by 3.

### Psychological tests

Psychological interviews were performed by a psychiatrist blinded to the CAG results. Subjects who met the inclusion criteria for the study completed the following psychological symptoms scales: Symptom Checklist 90 Revised (SCL-90-R), Beck Depression Inventory (BDI), and Beck Anxiety Inventory (BAI). The SCL-90-R is a revised version of the original SCL-90 (Derogatis et al.) scale, which is a psychiatric self-report inventory screening for general psychiatric symptomatology. This inventory focuses on nine dimensions: somatization, anxiety, obsessive-compulsive, depression, interpersonal sensitivity, psychoticism, paranoid ideation, hostility, and phobic anxiety^7^. Each of 90 items is scored on a five-point Likert scale from 0 (not at all) to 4 (extremely) according to the rate of occurence of each symptom over the last week. Global Severity Index (GSI) is a quantitative indicator concerning the respondent's current level of psychological distress and is calculated by summing the scores of nine dimensions and additional items, then dividing by the total number of responses. The realibility and validity of the Turkish version of SCL-90 has been analyzed by Dağ et al^8^.

The severity of depression was assessed using BDI, which is a 21 item self-report scale developed by Beck et al^9^. Items in the scale are rated from 0 to 3 in increasing order of severity. Item scores are totaled and can range from 0 to 63. Higher scores correlate with more severe depression. The pathologic cut-off value for the BDI score was determined to be 17 in the Turkish population, which reflects moderate and severe depressive states^10,11^. The validity and accuracy of the BDI in the Turkish population have been studied by Hisli et al^12^. Anxiety is measured using the 21-item self-reported BAI^13^. Each item is scored from 0 to 3 according to severity. Item scores are totaled and higher scores indicate higher anxiety levels. The pathologic cut-off value for the BAI score was determined to be 16 in the Turkish population; scores above this value reflect moderate to severe anxiety states^10,11^. The validity and realibility of the Turkish version of the BAI have been studied by Ulusoy et al^14^.

### Statistical Analyses

All data is presented as a mean ± SD for variables with normal distribution or a median [interquantile range] for variables with non-normal distribution. Categorical variables are reported as numbers and percentages. Continuous variables were checked for the normal distribution assumption using Kolmogorov-Smirnov statistics. Categorical variables were tested by Pearson's χ2 test and Fisher's Exact Test. Differences between patients and control subjects were evaluated using the Mann-Whitney U test or the Student t-test, when appropriate. The relation between numerical variables was identified using Pearson or Spearman's rho test. Multivariate linear regression analysis was performed to investigate the independent correlates of mean TFC. p-values were two sided and values < 0.05 were considered statistically significant. All statistical studies were carried out using Statistical Package for Social Sciences software (SPSS 16.0 for Windows, SPSS Inc., Chicago, Illinois).

## Results

Demographic, clinical, laboratory, echocardiographic, and angiographic characteristics of the 44 patients with CSF and 50 control subjects were summarized in Table 1. The two groups were similar in terms of age, sex, DM, HT, hyperlipidemia, alcohol consumption, smoking status, medications, heart rate, systolic blood pressure, low-density lipoprotein (LDL) cholesterol, and fasting plasma glucose levels. In addition, there was no significant difference in terms of monthly income and educational levels between the two groups (p = 0.93 and p = 0.55, respectively). TIMI frame counts were significantly higher in patients with CSF than those in controls (p = 0.01). In 24 (54%) of the cases, CSF was observed in one vessel, and in 20 (46%) of the cases, more than one vessel had CSF.

**Table 1:** Demographic, clinical, echocardiographic, laboratory, and demographic characteristics of the study population

**Characteristics**	**Control Group (n = 50)**	**Slow flow group (n = 44)**	**p value**
Age, years	53.8 ± 7.8	53.1 ± 10.4	0.73
Male, n (%)	27(54)	29 (66)	0.24
HT, n (%)	24(48)	20 (45)	0.80
Hyperlipiemia, n (%)	19 (38)	16 (36)	0.87
Diabetes, n (%)	9 (18)	8 (18)	0.98
Smoking, n (%)	27 (54)	20 (45)	0.41
Alcohol, n (%)	7 (14)	5 (11)	0.70
**Medications**			
ASA, n (%)	3 (6)	4 (9)	0.57
Beta Blocker, n (%)	3 (6)	2 (5)	0.76
ACEI, n (%)	6 (12)	5 (11)	0.92
ARB, n (%)	9 (18)	5 (11)	0.37
Calcium Channel Blocker, n (%)	7 (14)	6 (14)	0.95
Statin, n (%)	17 (34)	13 (30)	0.66
OAD, n (%)	6 (12)	5 (11)	0.92
Insulin, n (%)	5 (10)	6 (14)	0.58
Diuretic, n (%)	12 (24)	8 (18)	0.49
**Education level**			
Primary school, n(%)	23 (46)	25 (57)	
High school, n(%)	20 (40)	13 (29)	0.55
University, n(%)	7 (14)	6 (14)	
**Montiy income**			
Low, n(%)	27 (54)	25 (57)	
Intermediate, n(%)	20 (40)	17 (39)	0.93
High, n(%)	3 (6)	2 (4)	
BMI	25.6 ± 2.2	30.1 ± 6.4	0.01
Heart rate (bpm)	78 ± 8	76 ± 7	0.11
Systolic BP (mmHg)	117 ± 12	119 ± 13	0.85
LDL cholesterol (mg/dL)	108 ± 27	110 ± 31	0.87
Fasting plasma glucose (mg/dL)	100 ± 32	103 ± 35	0.71
LV ejection fraction (%)	60 ± 9	59 ± 8	0.61
**Total number of vessels with CSF**			
1 vessel , n(%)	-	24 (54)	-
≥1 vessel , n(%)	-	20 (45)	-
**TIMI frame counts**			
LAD	18.9 ± 1.1	27.7 ± 8.2	0.01
LCx	19.1 ± 1.7	24.5 ± 6.3	0.01
RCA	18.5 ± 1.3	24.9 ± 7.2	0.01

HT: hypertension; ACEI: angiotensin converting enzyme inhibitor; ARB: angiotensin receptor blocker; ASA: acetylsalicylic acid; BMI: body mass index; CSF: coronary slow flow; BP: blood pressure; LAD: left anterior descending; LCx: left circumflex; LDL: low-density lipoprotein; LV: left ventricular; n: number; OAD: oral antidiabetic; RCA: right coronary artery; TIMI: thrombolysis in myocardial infarction.

A comparison of psychiatric test results between the two groups is summarized in Table 2. In the CSF group, the BAI score was significantly higher than in controls (13 [18.7] vs 7.5 [7], p = 0.01). Twenty patients in the CSF group had a BAI score ≥ 16, which was significantly higher than the control group (8 cases) (p = 0.01). When the BDI test scores were compared between the two groups, subjects in the CSF group had significantly higher scores than those in control group (11 [14.7] vs. 6.5 [7], p = 0.01). The frequency of subjects with a BDI score ≥ 17 was higher in the CSF group (32% vs. 2%, p = 0.01). In addition, the median general symptoms index score was significantly higher in the CSF group (1.76 [0.81] vs 1.1[0.24], p = 0.01).

**Table 2:** Comparison of psychiatric tests results between CNF and CSF patients

**Characteristics**	**CNF (n = 50)**	**CSF (n = 44)**	**p value**
BAI	7.5 [7]	13 [18.7]	0.01
< 16, n (%)	42 (84)	24 (55)	0.01
≥ 16, n (%)	8 (16)	20 (45)	0.01
BDI	6.5 [7]	11 [14.7]	0.03
< 17, n (%)	46 (92)	30 (68)	0.01
≥ 17, n (%)	4 (8)	14 (32)	0.01
General Symptoms Index	1.1 [0.24]	1.76 [0.81]	0.01

BAI: Beck Anxiety Inventory; BDI: Beck Depression Inventory; CNF: coronary normal flow; CSF: coronary slow flow.

In univariate correlation analysis, mean TIMI scores were positively correlated with BAI (r = 0.56, p = 0.01), as were BDI scores (r = 0.47, p = 0.01) and the general symptom index (r = 0.65, p = 0.01); however, they were not correlated with age, educational level, monthly income, glucose, or LDL levels (Table 3). In addition, BMI was significantly correlated with mean TFC (r = 0.28, p = 0.01) but was not correlated with BAI (r = 0.16, p = 0.11), BDI (r = 0.08, p = 0.78), or GSI (r = 0.15, p = 0.18).

**Table 3:** Univariate correlation analysis of mean TIMI frame counts with study parameters

**Characteristics**	**Mean TIMI frame count**
BAI	r = 0.56; p = 0.01
BDI	r = 0.47; p = 0.01
General Symptoms Index	r = 0.65; p = 0.01
Age	r = 0.09; p = 0.36
BMI	r = 0.28; p = 0.01
Education level	r = -0.15; p = 0.15
Montly income	r = 0.08; p = 0.44
LDL	r = 0.02; p = 0.88
Glucose	r = 0.05; p = 0.67
EF	r = 0.09; p = 0.92

BAI: Beck Anxiety Inventory; BDI: Beck Depression Inventory; BMI: body mass index; LDL: low-density lipoprotein; EF: ejection fraction; TIMI: thrombolysis in myocardial infarction.

To investigate the independent determinants of mean TFC, we performed a multivariate linear regression analysis using a model adjusted for age, gender, and BMI. Results indicated that BMI (standardized β coefficient = 0.221; p = 0.01), BAI (standardized β coefficient = 0.546; p = 0.01), BDI (standardized β coefficient = 0.444; p = 0.01), and GSI (standardized β coefficient = 0.607; p = 0.01) were independently correlated with mean TFC. No correlation with age or gender was observed.

Correlation and subgroup analyses were performed to investigate the correlation of BAI, BDI, and GSI with atherosclerosis risk factors. In these analyses, BAI was not significantly correlated with age (r=0.09, p = 0.54), fasting glucose levels (r = -0.05, p = 0.64), LDL (r = -0.14, p = 0.19), or systolic blood pressure (r = 0.07, p = 0.72). BDI was not significantly correlated with age (r = 0.08, p = 0.41), fasting glucose levels (r = -0.11, p = 0.31), LDL (r = -0.14, p = 0.11), or systolic blood pressure (r = 0.17, p = 0.09). GSI was not significantly correlated with age (r = 0.03, p = 0.79), fasting glucose levels (r = -0.06, p = 0.65), LDL (r = -0.12, p = 0.26) or systolic blood pressure (r = 0.12, p = 0.24). In subgroup analysis, BAI, BDI, and GSI were not significantly different when compared between females and males (10 [14.9] vs. 8 [9.7], p = 0.26; 9 [13] vs. 8.5 [9.5], p = 0.18; 1.12 [0.92] vs. 1.38 [0.67], p = 0.46, respectively). In addition, BAI, BDI, and GSI were not significantly different between smokers and non-smokers (8 [10] vs. 10 [12], p = 0.21; 9 [8] vs. 9 [12], p = 0.49; 1.35 [0.57] vs. 1.21 [0.89], p = 0.57, respectively).

In total, 28 study participants (30%) had BAI scores ≥ 16 (Table 4). In these patients, total TFC and mean TFC were significantly higher (75.2 ± 17.5 vs. 62.4 ± 9.6, p = 0.01 and 25.1 ± 5.8 vs. 20.8 ± 3.2, p = 0.01, respectively). Age, gender, HT, DM, hyperlipidemia, smoking, alcohol consumption, educational status, monthly income, BMI, systolic blood pressure, fasting glucose, LDL cholesterol, and LVEF were similar among patients with BAI score < 16 and BAI score ≥ 16.

**Table 4:** Comparison of study parameters in patients with BAI score < 16 and ≥ 16

**Characteristics**	**BAI < 16 (n = 66)**	**BAI ≥ 16 (n = 28)**	**p value**
Age	53 ± 8.7	54.7 ± 9.8	0.42
Female Gender, n (%)	23(35)	15 (53)	0.06
HT, n (%)	32(48)	12 (43)	0.82
Hyperlipiemia, n (%)	23 (35)	12 (43)	0.48
Diabetes, n (%)	14(21)	3 (11)	0.38
Smoking, n (%)	36 (54)	11 (39)	0.36
Alcohol, n (%)	10 (15)	3 (11)	0.75
**Education level**			
Primary school, n(%)	31 (52)	19 (68)	
High school, n(%)	27 (41)	6 (21)	0.11
University, n(%)	8 (12)	3 (11)	
**Montly income**			
< 1500 TL, n (%)	36 (54)	16 (57)	
1500-3000 TL, n (%)	26 (39)	11 (39)	0.88
>3000 TL, n (%)	4 (6)	1 (3)	
BMI	27 ± 3.4	29.3 ± 7.7	0.07
Systolic BP (mmHg)	116 ± 10	118 ± 12	0.46
LDL cholesterol (mg/dL)	110 ± 28	113 ± 30	0.35
Glucose (mg/dL)	95 ± 33	103 ± 34	0.33
EF (%)	62 ± 4	59 ± 6	0.15
Mean TIMI FC	20.8 ± 3.2	25.1 ± 5.8	0.01
Total TIMI FC	62.4 ± 9.6	75.2 ± 17.5	0.01

BAI: Beck Anxiety Inventory; HT: hypertension; BMI: body mass index; BP: blood pressure; EF: ejection fraction; LDL: low-density lipoprotein; TIMI FC: thrombolysis in miyocardial infarction frame count; TL: Turkish Liras.

A comparison of study parameters in subgroups of patients classified as BDI score < 17 and ≥ 17 is depicted in Table 5. Overall, 76 patients (81%) had a BDI score < 17, whereas 18 patients (19%) had BDI scores ≥ 17. In both groups, age, gender, HT, DM, hyperlipidemia, smoking, alcohol, educational status, monthly income, BMI, systolic blood pressure, fasting glucose, LDL cholesterol, and LVEF were similar. Total TFC and mean TFC were significantly higher in subjects with a BDI score ≥ 17 than in those with a BDI score < 17. (78 ± 19 vs 63 ± 10; 25.9 ± 6.5 vs 21.1 ± 3.4, p = 0.01, respectively).

**Table 5:** Comparison of study parameters In patients with BDI score < 17 and ≥ 17

**Characteristics**	**BDI < 17 (n = 76)**	**BDI ≥ 17 (n = 18)**	**p value**
Age, years	53 ± 9.3	55.3 ± 8.2	0.36
Female gender, n (%)	28 (37)	10 (55)	0.15
HT, n (%)	37 (49)	7 (39)	0.60
Hyperlipiemia, n (%)	29 (38)	6 (33)	0.79
Diabetes, n (%)	13 (17)	4 (22)	0.73
Smoking, n (%)	41 (54)	6 (33)	0.19
Alcohol, n (%)	11 (14)	2 (11)	0.71
Education level			
Primary school, n (%)	40 (53)	10 (55)	
High school, n (%)	27 (35)	6 (33)	0.97
University, n (%)	9 (12)	2 (11)	
Montly income			
< 1500 TL, n (%)	41 (54)	11 (61)	
1500-3000 TL, n (%)	31 (41)	6 (33)	0.84
> 3000 TL, n (%)	4 (5)	1 (5)	
BMI	27 ± 5.4	28.7 ± 4.1	0.36
Systolic BP (mmHg)	116 ± 12	120 ± 11	0.21
LDL cholesterol (mg/dL)	110 ± 29	101 ± 27	0.23
Glucose (mg/dL)	100 ± 32	106 ± 42	0.47
EF (%)	62 ± 4	59 ± 5	0.14
Mean TIMI	21.1 ± 3.4	25.9 ± 6.5	0.01
Total TIMI	63 ± 10	78 ± 19	0.01

BDI: Beck Depression Inventory; HT: hypertension; BP: blood pressure; LDL: low-density lipoprotein; TIMI: Trombolysis in myocardial infarction; TLTurkish Liras; BMI: body mass index; EF: ejection fraction.

In a subgroup analysis of the 44 patients with CSF patients, those with CSF in more than one vessel had significantly higher BAI, BDI, and GSI scores than those with CSF restricted to one vessel (26 [16] vs. 8 [7.2], p = 0.01; 17 [14] vs 6 [1.1], p = 0.01; 2.1 [0.7] vs 1.4 [0.6], p = 0.01, respectively). In univariate correlation analysis, the number of vessels with CSF was positively correlated with BAI (r = 0.66, p = 0.01), BDI (r = 0.61, p = 0.01), and GSI (r = 0.59, p = 0.01).

## Discussion

Results of the present study indicate that patients with CSF had significantly increased levels of depression, anxiety, and overall psychological distress compared with patients having CNF.

The INTERHEART study is the largest trial conducted to date to investigate the correlation between stress and heart disease. This trial included 11,119 patients with MI from 52 countries. In this study, perceived stress and depression were shown to be important risk factors, which together accounted for 32.5% of the population with attributable risk for CAD. These findings suggest that these variables together were as important as smoking and more important than DM^15^. In a previous study, every 5-point increase in BDI score was associated with a 25%-30% increase in the risk of abnormal CAG findings or definitive CAD^10^. Furthermore, Shiozaki et al. demonstrated that depression emerging during the year after experiencing a MI is significantly associated with subsequent cardiovascular events in a 2.9-year follow-up for male patients^16^.

The prevalence of depression in the Turkish population is estimated to beabout 10%-20%^17^. Prior studies have estimated the prevalence of depression ranging from 20% to 40% in patients with CAD and have found that the presence of depression is associated with increased risk for adverse events^18^. In our study, 32% of patients with CSF were found to have major depressive disorder documented with a BDI score ≥17. Consistent with our results, Durmaz et al. have reported higher depression rates among patients with CSF (50% vs. 8%)^19^. Their study examined 90 patients and used the Hamilton Rating Scale (HAMD) to measure depression^19^. A total of 94 patients were included in our study but a different scale for depression (BDI) was used.

The pathophysiological mechanism between psychological distress and cardiovascular events has not been fully elucidated. Some of the proposed mechanisms are as follows: high sympathetic tone; increased cortisol and catecholamine; endothelial dysfunction; abnormal platelet activation, including enhanced platelet reactivity and release of platelet products (such as platelet factor 4 and b-thromboglobulin); augmented release of inflammatory markers; decreased heart rate variability; accelerated atherogenesis; and poor adherence^10,11^. We did not observe any correlation between level of anxiety or depression and atherosclerotic risk factors, which is similar to a prior report by Zafar et al^20^ Furthermore, no relationship between educational or monthly income (socioeconomic) status and depression or anxiety have been established.

Compared with the extensive literature on depression in patients with CAD, relatively few studies have examined the role of anxiety. Some studies have reported anxiety symptoms to be predictive of subsequent cardiac events, mortality, and in-hospital complications in patients with CAD, whereas others have found no association^21-23^. Martens et al^24^ have found that participants with baseline generalized anxiety disorder (GAD) had a greater rate of subsequent cardiovascular events than did participants without GAD. Vural et al^10^ found significant correlation between CAD and BAI scores. Todaro et al^25^ reported the lifetime prevalence of anxiety disorders to be 45.3% in patients with CAD. In addition, Durmaz et al^19^ investigated the relationship between anxiety and CSF. Unlike the other studies in the literature, their study utilized State-Trait Anxiety Inventory (STAI) for anxiety. They found that STAI scores were significantly higher in the CSF group. However, their study did not include a known cut-off value for anxiety; therefore, whether a difference was observed between the two groups in terms of the number of people with anxiety is unknown. As a result, no percentage was provided for anxiety^19^.

Our present study utilized the BAI scale to identify anxious people according to the cut-off value and found that 45.4% of patients with CSF had anxiety disorder; this was significantly higher than the control group. Previous studies have shown that obese and overweight people are more likely to experience anxiety and depression than those who are of normal weight^26^. However, comparison of the frequency of psychiatric disorders between overweight and obese people are controversial in the literature. In our study population, CNF patients were overweight, whereas patients with CSF were obese according to the mean BMI levels. As we did not intend to investigate correlation of obesity with psychiatric tests, our data may not be suitable to draw conclusions regarding obesity and psychiatric disorders; however, in our study, BMI was not correlated with BAI, BDI, and GSI scores.

GSI, which can be used as a summary of the SCL-90-R test, is designed to measure overall psychological distress. In our study, the GSI of patients CSF was 1.76, whereas the GSI of those subjects in the control group was 1.1 (p = 0.01), indicating that patients with CSF exhibit more psychological distress than those with normal coronary blood flow. Therefore, our study is the first study in the literature to use the SCL-90-R scale in patients with CSF.

Our study also found that patients with CSF in more than one coronary vessel had higher BAI, BDI, and GSI scores, which indicates the positive correlation between these scores and the extent and severity of CSF. Our study is the first study in the literature to report this correlation.

### Limitations

This study has several limitations that deserve mention. This is a single center, cross-sectional prospective study with relatively small sample size. In addition, we could not report the correlation of the study parameters with long-term outcomes in the study population.

## Conclusions

Although we could not draw a causal relationship, we observed higher levels of depression, anxiety, and overall psychological distress in patients with CSF. Further studies are needed to confirm our results and the importance of these findings in long-term outcomes and prognosis of patients with CSF.
